# The multiple roles of antibiotics and antibiotic resistance in nature

**DOI:** 10.3389/fmicb.2013.00255

**Published:** 2013-08-27

**Authors:** Fiona Walsh

**Affiliations:** Agroscope Changins WädenswilWädenswil, Switzerland

**Keywords:** antibiotic resistance, environment, clinical pathogens, soil, plasmids, intrinsic factor

There have been many calls for more information about the natural resistome and these have also highlighted the importance of understanding the environmental resistome in the preservation of antibiotics for the treatment of infections. However, to date there have been few studies which have investigated the roles of antibiotics and resistances outside of the clinical environment. This lack of data also highlights the difficulties faced by microbiologists in designing these experiments to produce meaningful data. Antibiotics and antibiotic resistance have most commonly been viewed in the context of human use and effects. However, both have co-existed in nature for millennia. Recently the roles of antibiotics and antibiotic resistance genes have started to be discussed in terms of functions other than bacterial inhibition and protection. This special topic has focused on both the traditional role of antibiotics as warfare mechanisms and their alternative roles and uses within nature.

The research topic starts with an introduction into antimicrobial resistance in medicine, its linkage to the global environmental microbiota and the many different roles of antibiotics and antibiotic resistance in nature, providing the background to the topic (Cantas et al., 2013). The following chapter discusses the idea that the understanding of antibiotic resistance implies expanding our knowledge on multi-level population biology of bacteria (Baquero et al., 2013). This brings with it inherent problems of designing experimental procedures and standards that can be used in many different microbiomes from human to soil and is discussed further by Walsh (Walsh, 2013). A number of models are proposed to study and understand the biological impact of selection and diversification of antibiotic resistance mechanisms, in particular using the β-lactamases as models (Galán et al., 2013; Patel and Bonomo, 2013; Popowska and Krawczyk-Balska, 2013). The β-lactamases constitute the most widespread mechanism of resistance, at least among pathogenic bacteria, with more than 1000 enzymes identified in the literature. We present some examples of the alternative functions for the multi-drug resistance mechanisms of efflux, that range from bacterial interactions with plant or animal hosts, to the detoxification of metabolic intermediates or the maintenance of cellular homeostasis and also the potential role of extracellular DNA in antibiotic resistance and virulence (Alvarez-Ortega et al., 2013). Bacterial responses to antibiotics may be concentration dependent and so we discuss the different types of interactions mediated by antibiotics and non-antibiotic metabolites as a function of their concentrations and speculate on how these may amplify the overall antibiotic resistance/tolerance and the spread of antibiotic resistance determinants in a context of poly-microbial community (Bernier and Surette, 2013; Lewenza, 2013; Sengupta et al., 2013). The use of antibiotics may also be regarded as pollution. Thus, the widespread use and abuse of antibiotic therapy has evolutionary and ecological consequences, some of which are only just beginning to be examined (Gillings, 2013).

These papers thoroughly review the many different aspects of antibiotic resistance and the roles of antibiotics in nature and link these to the emerging antibiotic resistances of particular importance to the treatment of infectious diseases.

## References

[B1] Alvarez-Ortega C. Olivares J. Martínez J. L. (2013). RND multidrug efflux pumps: what are they good for. Front. Microbiol. 4:7 10.3389/fmicb.2013.00007 23386844 PMC3564043

[B2] Baquero F. Tedim A. P. Coque T. M. (2013). Antibiotic resistance shaping multi-level population biology of bacteria. Front. Microbiol. 4:15 10.3389/fmicb.2013.00015 23508522 PMC3589745

[B3] Bernier S. P. Surette M. G. (2013). Concentration-dependent activity of antibiotics in natural environments. Front. Microbiol. 4:20 10.3389/fmicb.2013.00020 23422936 PMC3574975

[B4] Cantas L. Shah S. Q. A. Cavaco L. M. Manaia C. M. Walsh F. Popowska M. (2013). A brief multi-disciplinary review on antimicrobial resistance in medicine and its linkage to the global environmental microbiota. Front. Microbiol. 4:96 10.3389/fmicb.2013.00096 23675371 PMC3653125

[B5] Galán J. C. González-Candelas F. Rolain J. M. Cantón R. (2013). Antibiotics as selectors and accelerators of diversity in the mechanisms of resistance: from the resistome to genetic plasticity in the β-lactamases world. Front. Microbiol. 4:9 10.3389/fmicb.2013.00009 23404545 PMC3567504

[B6] Gillings M. R. (2013). Evolutionary consequences of antibiotic use for the resistome, mobilome and microbial pangenome. Front. Microbiol. 4:4 10.3389/fmicb.2013.00004 23386843 PMC3560386

[B7] Lewenza S. (2013). Extracellular DNA-induced antimicrobial peptide resistance mechanisms in *Pseudomonas aeruginosa*. Front. Microbiol. 4:21 10.3389/fmicb.2013.00021 23419933 PMC3572637

[B8] Patel G. Bonomo R. A. (2013). “Stormy waters ahead”: global emergence of carbapenemases. Front. Microbiol. 4:48 10.3389/fmicb.2013.00048 23504089 PMC3596785

[B9] Popowska M. Krawczyk-Balska A. (2013). Broad-host-range IncP-1 plasmids and their resistance potential. Front. Microbiol. 4:44 10.3389/fmicb.2013.00044 23471189 PMC3590792

[B10] Sengupta S. Chattopadhyay M. K. Grossart H. P. (2013). The multifaceted roles of antibiotics and antibiotic resistance in nature. Front. Microbiol. 4:47 10.3389/fmicb.2013.00047 23487476 PMC3594987

[B11] Walsh F. (2013). Investigating antibiotic resistance in non-clinical environments. Front. Microbiol. 4:19 10.3389/fmicb.2013.00019 23423602 PMC3573686

